# pH and Redox Dual-Sensitive Covalent Organic Framework Nanocarriers to Resolve the Dilemma Between Extracellular Drug Loading and Intracellular Drug Release

**DOI:** 10.3389/fchem.2020.00488

**Published:** 2020-06-26

**Authors:** Chaoyu Wang, Huiming Liu, Shuai Liu, Zhijun Wang, Jianhua Zhang

**Affiliations:** ^1^School of Chemistry and Chemical Engineering, Mudanjiang Normal University, Mudanjiang, China; ^2^Key Laboratory of Systems Bioengineering of the Ministry of Education, Department of Polymer Science and Engineering, School of Chemical Engineering and Technology, Tianjin University, Tianjin, China; ^3^Tianjin Key Laboratory of Membrane Science and Desalination Technology, Tianjin University, Tianjin, China; ^4^People's Hospital of Yujiang District, Yingtan, China

**Keywords:** covalent organic frameworks, nanocarriers, dual-sensitive, drug delivery, doxorubicin

## Abstract

Cancer poses a serious threat to human health. To enhance the efficacy of tumor chemotherapy, it is urgent to develop novel and effective nanocarriers with the ability to efficiently load and deliver anticancer drugs. Covalent organic frameworks (COF)-based nanocarriers (CONs) have exhibited great potential for drug loading due to their porous structure and high surface area. However, the function of tumor intracellular-triggered drug release has barely been integrated. Herein we first synthesized a kind of hydrazide and disulfide bonds containing building block (4,4'-Dihydrazide diphenyl disulfide, **DHDS**), which was used to develop a PEGylated pH and redox dual-sensitive CONs (denoted HY/SS-CONs) for efficiently loading and delivering doxorubicin (DOX). The obtained HY/SS-CONs can achieve a very high loading content of DOX and very low premature leakage at physiological condition. However, under tumor intracellular microenvironment, HY/SS-CONs with acid-cleavable hydrazone bonds, and GSH-exchangeable disulfide bonds will undergo rapid disintegration, and efficiently release DOX to kill tumor cells. The COFs-based dual-sensitive nanocarriers provide a promising solution to the dilemma of extracellular drug loading and tumor intracellular drug release.

## Introduction

Cancer has posed a lethal threat to humans around the world. As a major treatment modality, chemotherapy by use of anticancer drugs has achieved obvious success in prolonging patient survival. Nevertheless, the clinical efficacy of chemotherapy is still far from satisfactory and severely limited by the intrinsic limitations of anticancer drugs, including poor stability, low water solubility, nonspecific drug distribution, and terrible side effects to healthy tissues (Chabner and Roberts, 2005). Over the past several decades, a great number of nanoparticulate drug delivery systems, such as liposomes (Bozzuto and Molinari, 2015), polymer-based micelles (Kamaly et al., 2016), inorganic and metal nanocarriers (Huang et al., 2011; Zhang H. et al., 2018; Vines et al., 2019; Xu et al., 2019), and nanogels (Zhang et al., 2015; Yu et al., 2018; Zhao X. et al., 2018), as well as hybrid nanoplatforms (Raemdonck et al., 2014), have been designed and applied to overcome the drawbacks associated with conventional drug formulations. Apparently, these nanocarriers have demonstrated their ability to protect the drugs from premature degradation, increase the drug solubility, improve the drug accumulation within tumors, and enhance the tumor's intracellular distribution of drug, thus leading to a higher efficiency and lower toxicity. However, the potential of these nanocarriers is far from being fully exploited and there are still some issues and improvements that need to be addressed (Mura et al., 2013; Dawidczyk et al., 2014; Blanco et al., 2015; Shi et al., 2017).

One of major limitations for most traditional nanocarriers is their low capacity for loading drugs (Blanco et al., 2015; Shi et al., 2017). For example, the drug loading of conventional liposomes and polymeric micelles is often below 1 and 5%, respectively. The low drug loading content always results in insufficient efficacy. Therefore, to achieve an ideal therapeutic outcome, it demands an increase in dosage and/or frequency of administration, often leading to a higher risk of adverse effects caused by the drugs and pharmaceutical excipients. In addition, some nanocarriers, especially those derived from assembly process, often suffer from premature drug leakage, thus causing poor therapeutic efficacy and severe systemic toxicity (Blanco et al., 2015; Shi et al., 2017). Apparently, it is imperative to develop more sophisticated nanocarriers with the ability to effectively load drugs and stably retain the payloads in the bloodstream.

During the last decade, covalent organic frameworks (COFs), as a class of newly emerged crystalline porous polymers, have attracted enormous attention. COFs were linked by dynamic covalent bonds such as imine, imide, azine, hydrazone, and boronate ester, and made from building blocks that were mainly consisted of lightweight elements (H, B, C, N, and O) (Diercks and Yaghi, 2017). COFs possess some desirable and unique features, including tunable pore geometry, large surface area, outstanding crystallinity, intrinsic adaptability, and excellent flexibility in molecular architecture, and functional design, thus exhibiting great potential for various applications (Sakaushi and Antonietti, 2015; Waller et al., 2015; Kandambeth et al., 2018; Song et al., 2019; Zhao et al., 2019). Recently, these porous frameworks opened up a new avenue for exciting opportunities in biomedical and pharmaceutical fields, especially for drug delivery (Fang et al., 2015; Bai et al., 2016; Vyas et al., 2016; Mitra et al., 2017; Wu and Yang, 2017; Hashemzadeh and Raissi, 2018; Zhao F. et al., 2018; Liu et al., 2020). The COFs as drug carriers have demonstrated some unique advantages. Some COFs-based drug delivery systems were proven to possess high loading capacity and minimized drug leakage due to the porous structure, high surface area, and π-π stacking interactions between COFs and drugs (Liu H. et al., 2019; Wang et al., 2019). However, the high and stable drug loading usually signifies slow drug release, leading to insufficient drug accumulation at the target site. These intrinsic limitations often cause treatment failure and even multidrug resistance, which severely impeded the practical applications of COFs-based carriers in anticancer drug delivery (Blanco et al., 2015; Fang et al., 2015; Shi et al., 2017; Zhao F. et al., 2018).

The physiological pH of blood and normal tissues is about 7.4, but the tumor intracellular endo/lysosomal pH ranges from 4.0 to 6.5. In addition, the glutathione concentration (GSH, 2–10 mM) within tumor cells is substantially higher than extracellular levels (1–2 μM) in plasma. The difference in pH value and GSH level between tumor intracellular and extracellular microenvironment is advantageous for the specifically targeted and controlled drug release (Cheng et al., 2011; Lee et al., 2013; Mura et al., 2013; Blanco et al., 2015; Du et al., 2018). Moreover, pH- and/or GSH-stimulus-sensitive nanocarriers have been widely reported for the controllable drug release in tumor cells (Zhang et al., 2013; Deng et al., 2015; Ma et al., 2018; Liu F. et al., 2019), which are based on the tumor cell microenvironment-sensitive bonds, such as acid-cleavable hydrazone, or acetal bonds and GSH-exchangeable disulfide bonds. Nevertheless, the function of intracellular-triggered drug release has barely been integrated into the COFs-based drug delivery systems.

**Scheme 1:**
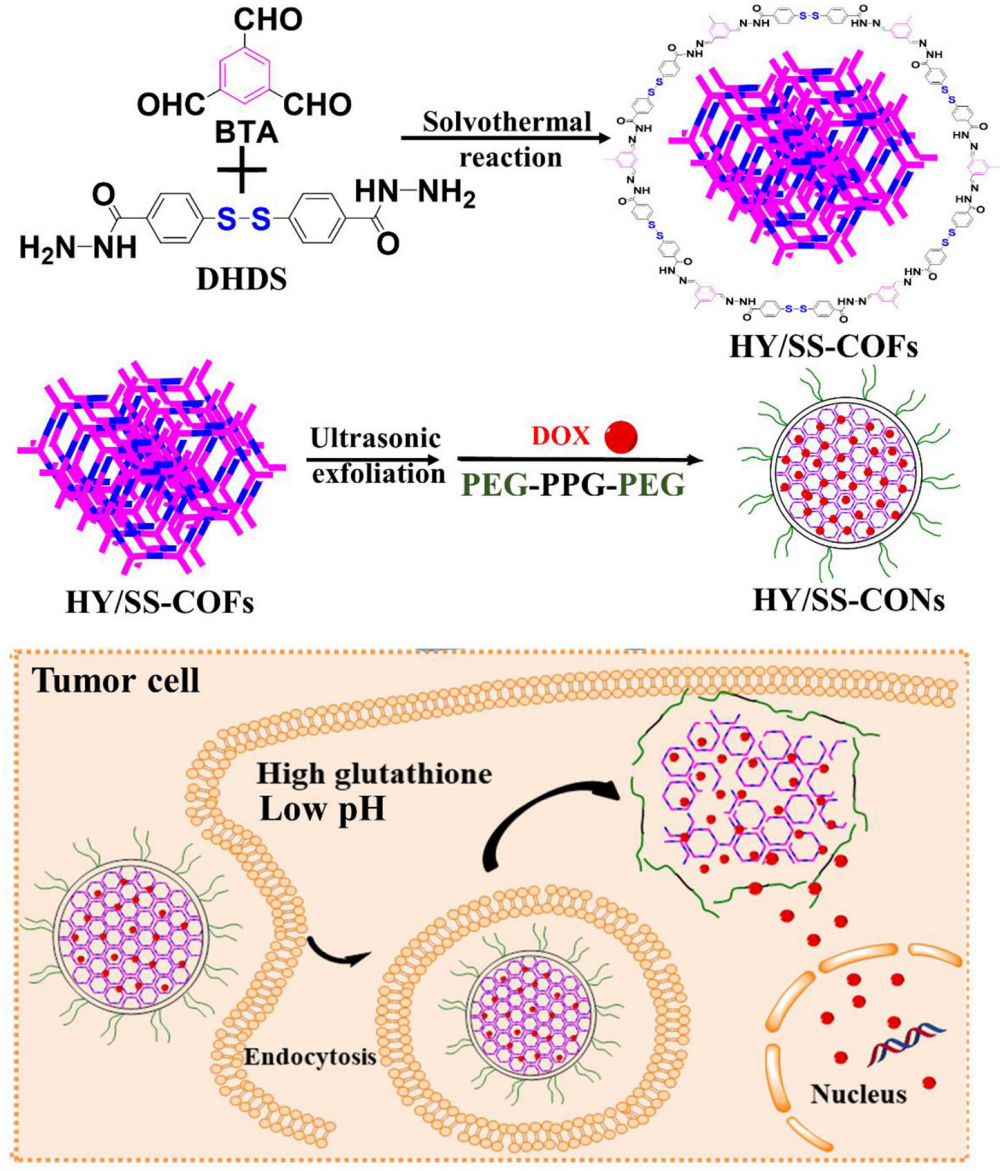
Schematic illustrations of the preparation process of DOX-loaded HY/SS-CONs and their intracellular responsive drug release.

In this study, we designed and prepared a kind of pH and redox dual-sensitive covalent organic framework nanocarriers (CONs) for efficiently loading and delivering doxorubicin (DOX). As shown in Scheme 1, we first synthesized a kind of new building block (4,4'-Dihydrazide diphenyl disulfide, **DHDS**), which has both two hydrazide bonds and a disulfide bond. Then, the hydrazone and disulfide-containing COFs (**HY/SS-COFs**) were prepared by use of a versatile Schiff-base reaction between DHDS and benzene-1,3,5-tricarbaldehyde (**BTA**). After ultrasound exfoliation and co-assembly with Poloxamer 188 (PEG-PPG-PEG, an FDA-approved pharmaceutic adjuvant), the pH and redox dual-sensitive nanocarriers (denoted as **HY/SS-CONs**) were prepared. HY/SS-CONs can efficiently load DOX due to their porous structure and high surface area, as well as the hydrophobic and π-π stacking interactions between HY/SS-CONs and DOX. Moreover, HY/SS-CONs can undergo structural disintegration under tumor cell microenvironment and rapidly release the payload in response to low pH and high GSH level in tumor cells. The PEG chains of Poloxamer 188 anchored on the outer surface of HY/SS-CONs can improve the dispersion stability in water and long-circulation capacity. The designed HY/SS-CONs have the ability to balance extracellular drug loading and intracellular drug release, thus holding great promise for anticancer drug delivery.

## Materials and Methods

### Materials

1,3,5-benzenetricarboxaldehyde (BTA), 4-sulfhydryl benzoic acid methyl ester (SBME), and glutathione (GSH) were purchased from Aladdin Reagent Limited Company (Shanghai, China). Poloxamer 188 with an average molecular weight of about 8,400 daltons was purchased from Jiaxing Sicheng Chemical Co., Ltd. (Zhejiang, China). Doxorubicin (DOX) was purchased from Meilunbio Company (Dalian, China). All other reagents were commercially available without further purification.

### Synthesis and Characterizations of 4,4'-Dihydrazide Diphenyl Disulfide (DHDS)

The hydrazide groups were firstly introduced to prepare 4-sulfhydryl benzoic acid hydrazide (SBH). Briefly, 4-sulfhydryl benzoic acid methyl ester (SBME) (1 g, 5.6 mmol) was dissolved in anhydrous methanol, and then the hydrazine hydrate solution (7 ml, 80%) was dropwise added. After completely mixing, the reaction was performed for 24 h at 60°C under string. After rotary evaporation to remove the solution and recrystallization, the faint yellow solid SBH was obtained. Subsequently, the SBH (0.57 g, 3.4 mmol) and iodine (0.44 g, 1.7 mmol) were dissolved in anhydrous ethanol. After dropwise addition of triethylamine (1.5 mL, 10.2 mmol), the reaction was performed for 24 h at room temperature. Sodium thiosulfate solution (10%) was added into above mixture to remove the excessive unreacted iodine. After adjusting the pH value to about 5.0 using 0.01 M HCl, the yellow solid DHDS was obtained after filtration, washing by water, and drying in vacuum. The molecular structure of DHDS was confirmed by ^1^H-NMR and electrospray ionization mass spectrometry (ESI-MS). ^1^H-NMR spectra were recorded in *d*-DMSO using Varian Inova-500M spectrometer instrument (Varian Inc. Palo Alto, USA). The ESI-MS was a single-quadrupole VG-platform spectrometer with MassLynx version 3.1. The ESI-MS data were obtained in positive-ion mode. Sodium formate was added to the solvent to enhance the electrospray ion current. The skimmer voltage was 40 V.

### Synthesis and Characterizations of HY/SS-COFs and HY/SS-CONs

The HY/SS-COFs described in Scheme 1 were synthesized by solvothermal synthesis methods. Briefly, BTA (32.4 mg, 0.2 mmol) and DDS (100.2 mg, 0.3 mmol) were completely dissolved in the mixture of 1,4-dioxane and mesitylene (10 mL, 1:3 in voL) in a Schlenk tube. The mixture was degassed by three freeze-evacuate-thaw cycles and then heated at 120°C in a thermostatic oil bath for 72 h. After centrifugation, repeated washing by tetrahydrofuran, and vacuum drying, the yellow solid product (HY/SS-COFs) were obtained. After sonication exfoliation for about 10 min, HY/SS-COFs were broken into CONs with size of 100 nm. Sonication was carried out with a Skymen JM-03D-28 ultrasonic cleaner with a power of 120 W (Skymen Cleaning Equipment Co., Ltd, Shenzhen, China). The DOX was encapsulated into CONs by the equilibrium adsorption and diffusion. Briefly, CONs (100 mg) were dispersed into n-hexane solution (10 mL) of DOX (30 mg/mL) and then slowly stirred for 24 h at room temperature under dark conditions. The DOX-loaded CONs were obtained by centrifugation, repeated washing by n-hexane, and vacuum drying. To prepare drug-free and drug-loaded PEGylation HY/SS-COFs, the drug-free and drug-loaded CONs (100 mg) were dispersed into DMSO solution (10 mL) containing Poloxamer 188 (2 g). After sonication for about 5 min, the mixture was dropwise added into deionized water (50 mL) under stirring. After full dispersion for about 24 h, DMSO and free Poloxamer 188 was removed by dialysis for 24 h in water at room temperature with a dialysis bag (MWCO = 50000 g/mol). Finally, the drug-free and drug-loaded HY/SS-COFs were obtained after lyophilization.

The size and morphology of above HY/SS-COFs and HY/SS-CONs under different conditions were measured by dynamic light scattering (DLS, Zetasizer Nano ZS90), scan electron microscopy (SEM, Hitachi S-4800), and/or transmission electron microscope (TEM, JEOL JEM-2100F). The powder X-ray diffraction (PXRD) detections were carried out using a Bruker D8 Focus diffractometer (Bruker AXS, Germany). The pore size distribution and surface area were determined by nitrogen adsorption/desorption experiment with a Micrometric ASAP 2010 instrument. The specific area was calculated using Brunauer-Emmett-Teller (BET) model and the pore size was obtained from Barrett-Joyner-Halenda (BJH) method. Thermogravimetry (TGA) was measured on a NETZSCH STA 449 C thermogravimetric analyzer from 0 to 800°C at a heating rate of 10°C/min in nitrogen flow. Fourier transform infrared spectroscopy (FTIR) was carried out with a Perkin-Elmer FTIR spectrometer (Perkin Elmer, Waltham, USA).

The DOX loading content as well as *in vitro* release behavior under different conditions (pH 7.4 and pH 5.0 with or without 10 mM GSH) at 37°C were investigated. The amount of DOX in DOX-loaded HY/SS-CONs was determined by a WFZ-26A UV/Vis spectrophotometer at 480 nm. Typically, DOX-loaded HY/SS-CONs (1 mg) were dispersed in distilled water (10 mL) and incubated at room temperature for 24 h after adding 1.0 mol/L HCl (1 mL) with 10 mM GSH. After the complete disintegration of HY/SS-CONs, the concentration of DOX was analyzed by UV/Vis spectrophotometer and calculated using a calibration curve of different DOX concentrations in an identical solvent mixture. To test the drug release behaviors, DOX-loaded HY/SS-CONs solution (1.0 mg/mL) was sealed in a dialysis bag with a MWCO of 3500. The dialysis bag was immersed in a screw capped bottle containing appropriate buffer medium (20 mL) in a shaking bath at 37°C. At regular time intervals, 5.0 mL of the release solution was removed and the same volume of fresh buffer solution was added to maintain a constant volume of the released medium. The amount of the released drug was determined by UV/Vis spectrophotometer at 480 nm. All experiments were carried out in triplicate.

The dispersion stability and GSH and/or pH-triggered size and morphological transitions of HY/SS-CONs were investigated as below. Drug-loaded HY/SS-CONs (0.5 mg) were dispersed in PBS (5.0 mL) at pH 7.4. The size changes were determined after predesignated incubation time by DLS measurement and Tyndall phenomenon. To investigate the hemodynamic stability, the particle size of HY/SS-CONs (0.1 mg/mL) in pH 7.4 PBS containing 5% bovine serum albumin (BSA) at 37°C was measured at fixed time points. The GSH and/or pH-sensitivity of HY/SS-CONs was evaluated by DLS measurement of size change with time of drug-loaded HY/SS-CONs in PBS pH 5.0 with 10 mM GSH.

### *In vitro* Cytotoxicity Test and Cellular Uptake Evaluation

The MTT assay was utilized to evaluate the cellular toxicity of free DOX, drug-free, and drug-loaded HY/SS-CONs. HepG2 cells were seeded into a 96-well plate with a density of 1 × 10^5^ cells per well and incubated for 24 h at 37°C with 5% CO_2_. Then the Dulbecco's Modified Eagle's medium (DMEM) was discarded and the cells were washed twice with pH 7.4 PBS solution. Various concentrations of drug-free HY/SS-CONs in PBS at pH 7.4 were added into the wells for co-culture with the cells for 24 h. After incubation, the MTT assay was carried out to determine the cell viability. According to similar protocols, the cellular toxicity toward HepG2 cells of free DOX and DOX-loaded HY/SS-CONs with an equivalent DOX concentration was investigated. HepG2 cells were incubated for 24 h with free DOX and DOX-loaded HY/SS-CONs in PBS at pH 7.4, respectively. All experiments were carried out in quintuplicate to determine mean values and standard deviations. To investigate the cellular uptake of HY/SS-CONs, HepG2 cells were seeded into a confocal microscopic dish at a density of 1 × 10^5^ per dish and incubated in DMEM medium at 37°C for 24 h. And then the culture medium was replaced by PBS solution containing pure DOX or DOX-loaded HY/SS-CONs at a concentration of 10 μg/mL. After incubation for 4 h, the cells were washed with PBS solution three times and then stained with DAPI for 5 min. After repeated wash with PBS solution, cellular endocytosis was evaluated using a confocal laser scanning microscope (TCS SP8, Leica).

## Results and Discussion

### Synthesis and Characterizations of DHDS

**Figure 1:**
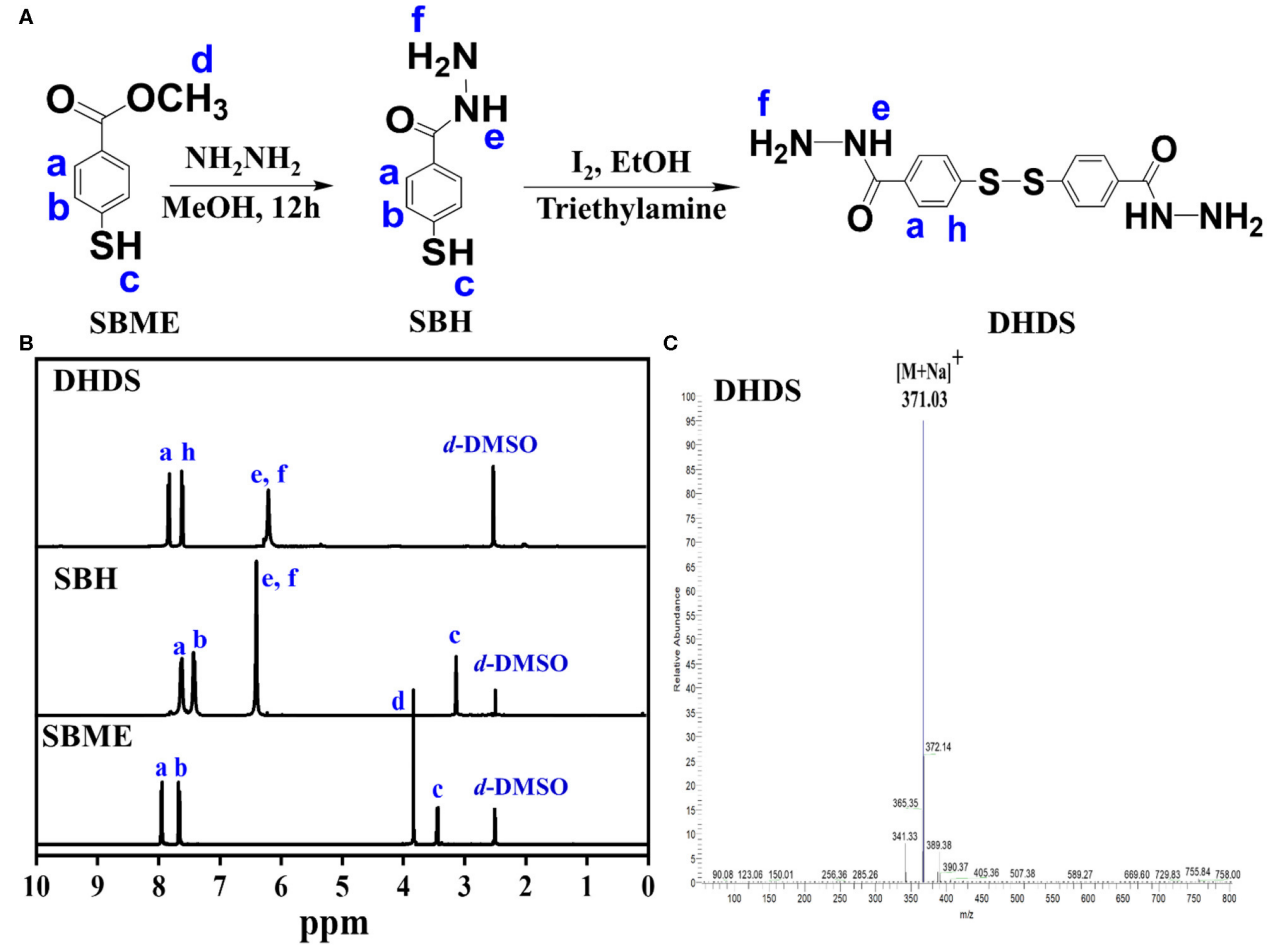
Synthesis and characterization of DHDS. **(A)** Synthesis process; **(B)** ^1^HNMR spectra of SBME, SBH, and DHDS; **(C)** ESI-MS spectrum of DHDS.

DHDS has both two hydrazide bonds and a disulfide bond, which can act as building blocks for construction of pH and redox sensitive COFs with acid-cleavable hydrazone and GSH-exchangeable disulfide bonds. The synthesis process of DHDS was shown in Figure 1A. First, the precursor SBME was hydrazide-functionalized, then the obtained SBH was oxidized to produce DHDS. ^1^HNMR was used to monitor the chemical transformations of SBME and SBH in the reaction process and determine the composition and structure of DHDS. As shown in Figure 1B, the ^1^HNMR spectrum of DHDS exhibited the characteristic peaks of phenyl ring at 7.7–8.0 ppm (**a**, **h**) and the characteristic peaks corresponding to hydrazide at about 6.2 ppm (**e**, **f**). In addition, the disappeared peaks of the methyl protons (**d**) at about 3.8 ppm of SBME and the disappeared peak of sulfhydryl protons (**c**) of SBH can be used to further confirm the formation of DHDS. Moreover, after the integration of the respective peak or peak groups from left to right in the ^1^HNMR spectrum of DHDS, it can be found that the area of the peak at 6.2 ppm (**e**, **f**) is 1.5 times greater than the area of the peak at 7.9 ppm (**a**) or 7.7 ppm (**h**). This result is very close to its theoretical value, thus indicating the molecular structure of DHDS. The ESI-MS was used to further confirm the molecular structure of DHDS, as shown in Figure 1C. The mass spectrum of DHDS was dominated by the protonated ions [M+Na]^+^ at m/z 371.03 and exhibited negligible fragmentation. The observed molecular mass is in excellent agreement with the theoretical value of DHDS, demonstrating the successful synthesis of DHDS.

### Synthesis and Characterization of HY/SS-COFs

**Figure 2:**
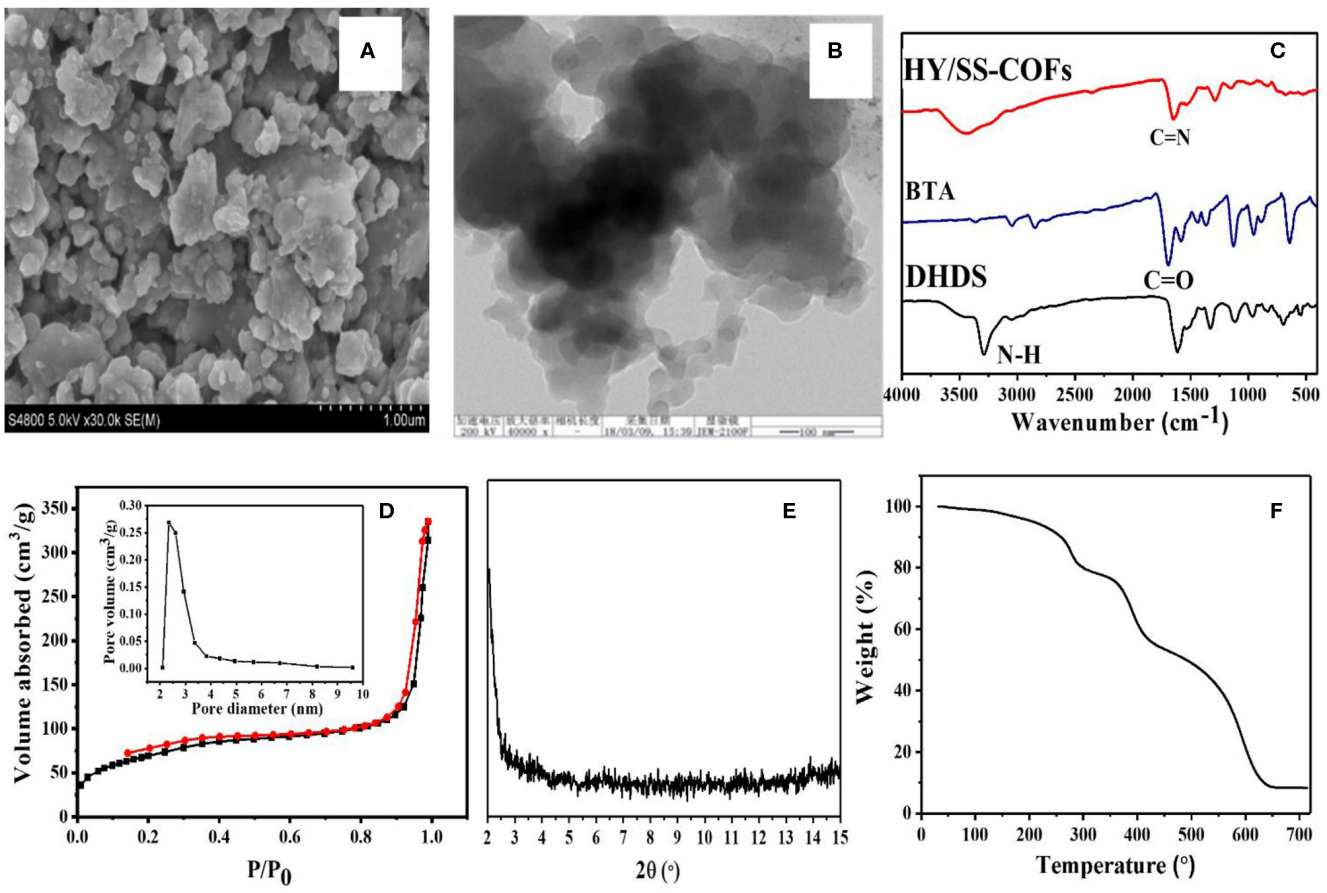
Characterizations of HY/SS-COFs. **(A)** SEM image and **(B)** TEM image; **(C)** FTIR spectra of HY/SS-COFs, BTA, and DHDS; **(D)** Nitrogen adsorption and desorption isotherms. The inset shows pore size of HY/SS-COFs; **(E)** PXRD pattern; **(F)** TGA curve.

A series of COFs has demonstrated great potential as a promising platform for effective loading and delivery of various drugs, because of their unique and attractive features (Fang et al., 2015; Bai et al., 2016; Vyas et al., 2016; Mitra et al., 2017; Wu and Yang, 2017; Hashemzadeh and Raissi, 2018; Zhao F. et al., 2018; Liu et al., 2020). In particular, the hydrazone-linked COFs based on Schiff-base chemistry exhibited a high stability under physiological conditions but acid-cleavable property within tumoral acid microenvironment, providing some unique advantages for biomedical, and pharmaceutical applications (Segura et al., 2016; Zhao F. et al., 2018). The hydrazone and disulfide bonds-bearing HY/SS-COFs were prepared by use of a solvothermal Schiff-base reaction between commercially available BTA and DHDS as building blocks. The obtained HY/SS-COFs were characterized in detail, as shown in Figure 2. SEM image of HY/SS-COFs indicates the shapes of HY/SS-COFs are irregular slices consisted of small flake-like structures (Figure 2A). A further inspection of the structures by TEM in Figure 2B indicated that the HY/SS-COFs have a spherical nanoparticle-like morphology. The FTIR spectra of HY/SS-COFs, BTA, and DHDS were shown in Figure 2C. It can be seen that DHDS showed the characteristic peaks, such as amines (-NH-) at about 3,320 cm^−1^ and disulfide (-S-S-) at about 510 cm^−1^. On inspection of the FTIR spectrum, DHDS presented a characteristic peak of -C-N- bonds at about 1,420 cm^−1^, which further indicated the successful preparation of DHDS. Compared with the FTIR spectrum of DHDS, the presence of a weak imine C=N stretch at about 1,620 cm ^−1^ was observed in the FTIR spectrum of HY/SS-COFs. Furthermore, the absence of the aldehydic C-H and C=O stretching vibrations of BTA further confirmed the formation of HY/SS-COFs. Nitrogen adsorption/desorption analysis of HY/SS-COFs was shown in in Figure 2D. The results indicated that HY/SS-COFs possess a very high surface area of 328 m^2^/g and narrow pore size distribution with pore size of about 2.5 nm. PXRD indicates the formation of HY/SS-COFs had a poor crystalline structure (Figure 2E). The thermal stability of HY/SS-COFs was monitored using TGA, as shown in Figure 2F. The result indicates HY/SS-COFs have excellent thermal stability and their decomposition behavior occurs at nearly over 300°C. About 10% of the weight loss was observed at the temperature lower than 300°C. This weight loss below 300°C may be due to the loss of the absorbed solvent or monomers in the pores of cores, as well as the unreacted hydrazone or aldehyde groups at the termini of COFs (Ma et al., 2016; Xiong et al., 2020). Subsequently, HY/SS-COFs showed a sharp weight loss profile at the temperature higher than 400°C and ~60% weight loss was observed between 400 and 700°C. This significant weight loss can be attributed to the structural destruction and decomposition of COFs.

### Preparation and Characterization of HY/SS-CONs

**Figure 3:**
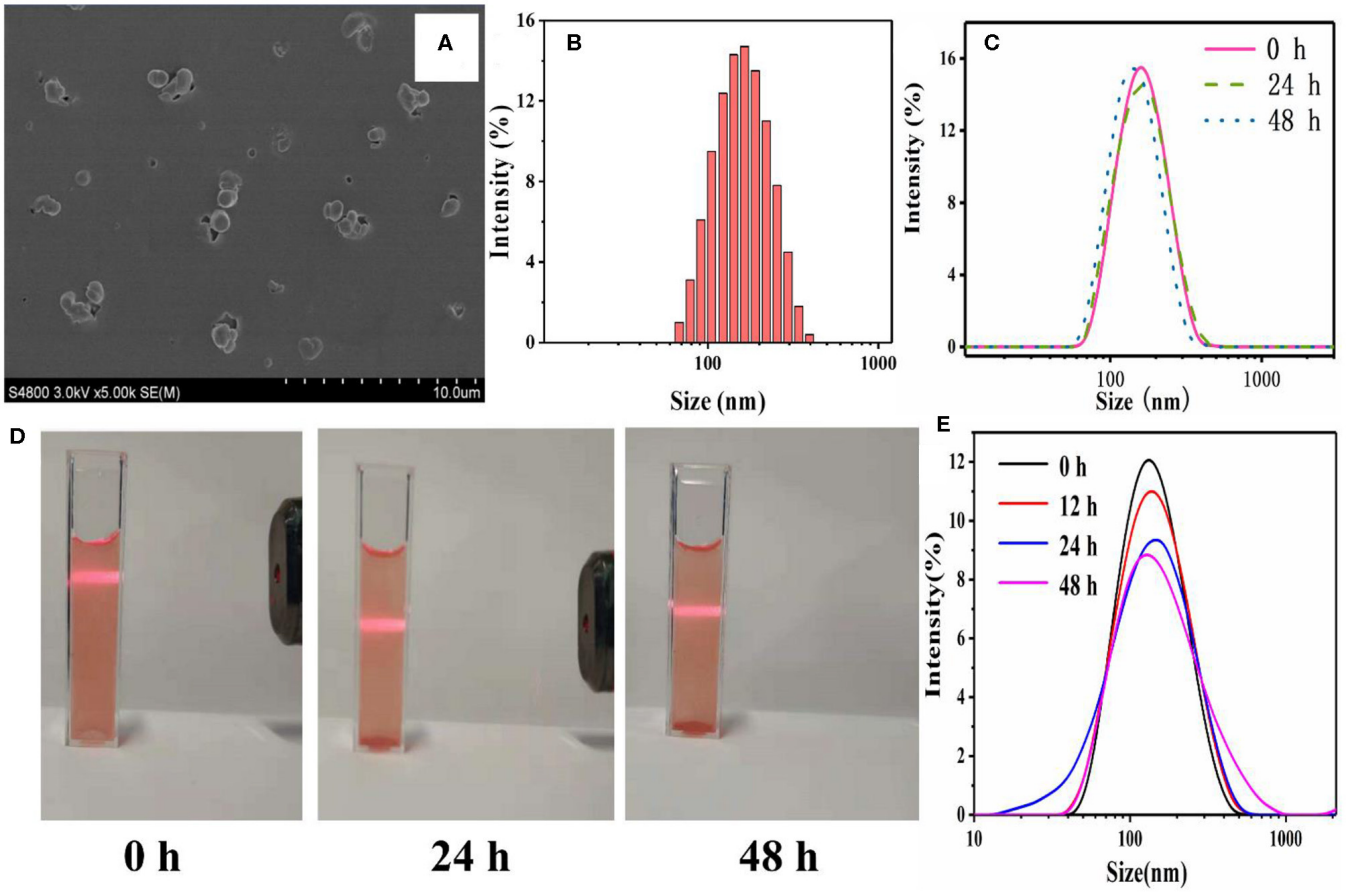
Characterizations of HY/SS-CONs. **(A)** SEM image and **(B)** DLS pattern; **(C)** Storage stability of DOX-loaded HY/SS-CONs at pH 7.4; **(D)** Tyndall phenomena of DOX-loaded HY/SS-CONs at pH 7.4; **(E)** Hemodynamic stability of HY/SS-CONs in PBS pH 7.4 containing 5% BSA at 37°C.

COFs had great potential for drug loading and delivery. However, before entering into the blood circulation system, the COFs have to be exfoliated into nanoscale platelets and be surface-modified (such as PEGylation) in order to overcome the physicochemical and physiological stability limitations (Zhao F. et al., 2018). The ultrasonic exfoliation was widely used to exfoliate COFs. Moreover, the co-assembly of nanoscale COFs with PEG-containing amphiphilic polymers has been proven to act as an efficient approach to anchor PEG chains onto the outer surface of COFs (Zhang G. et al., 2018; Liu F. et al., 2019; Wang et al., 2020). The PEGylated nanoscale COFs, [i.e., HY/SS-CONs, were readily prepared by through the co-assembly of FDA-approved Poloxamer 188 (a kind of PEG-containing amphiphilic polymer, PEG-PPG-PEG), with the nanoscale HY/SS-COFs formed by ultrasonic exfoliation]. Due to the hydrogen bond and hydrophobic interactions, the hydrophobic PPG chain of Poloxamer 188 chains can adsorbed onto the surface of nanoscale HY/SS-COFs to generate HY/SS-CONs. After effective removal of excess Poloxamer 188 by dialysis, the HY/SS-CONs were obtained and characterized. As shown in Figure 3A, the SEM image of HY/SS-CONs clearly indicates its nearly spherical morphology. The particle sizes of HY/SS-CONs in SEM image is about 120 ± 20 nm. The hydrodynamic size of HY/SS-CONs was tested by DLS and shown in Figure 3B, indicating a size of about 140 nm with relatively high monodispersity. The dispersion stability is an important factor for the application of nanocarriers. As shown in Figure 3C, a significant size fluctuation was not observed for about 48 h, indicating a high long-term stability. The dispersion stability of HY/SS-CONs in PBS pH 7.4 was further confirmed by the stable Tyndall phenomena in Figure 3D indicated that F68@SS-COFs can be stably dispersed. The excellent stability of HY/SS-CONs can be ascribed to the combined effect of the steric stabilization of PEGylated shell preventing flocculation and the steady CONs core ensuring integrity of nanostructure. The size change as a function of incubation time for HY/SS-CONs in PBS pH 7.4 containing 5% BSA at 37°C is shown in Figure 3E. No significant change in size can indicate the absence of aggregation or sedimentation, mainly due to the steric stabilization of PEGylated shell to minimize interactions with proteins and maintain the hemodynamic stability.

### Drug Loading and Dual-Sensitive Release

**Figure 4:**
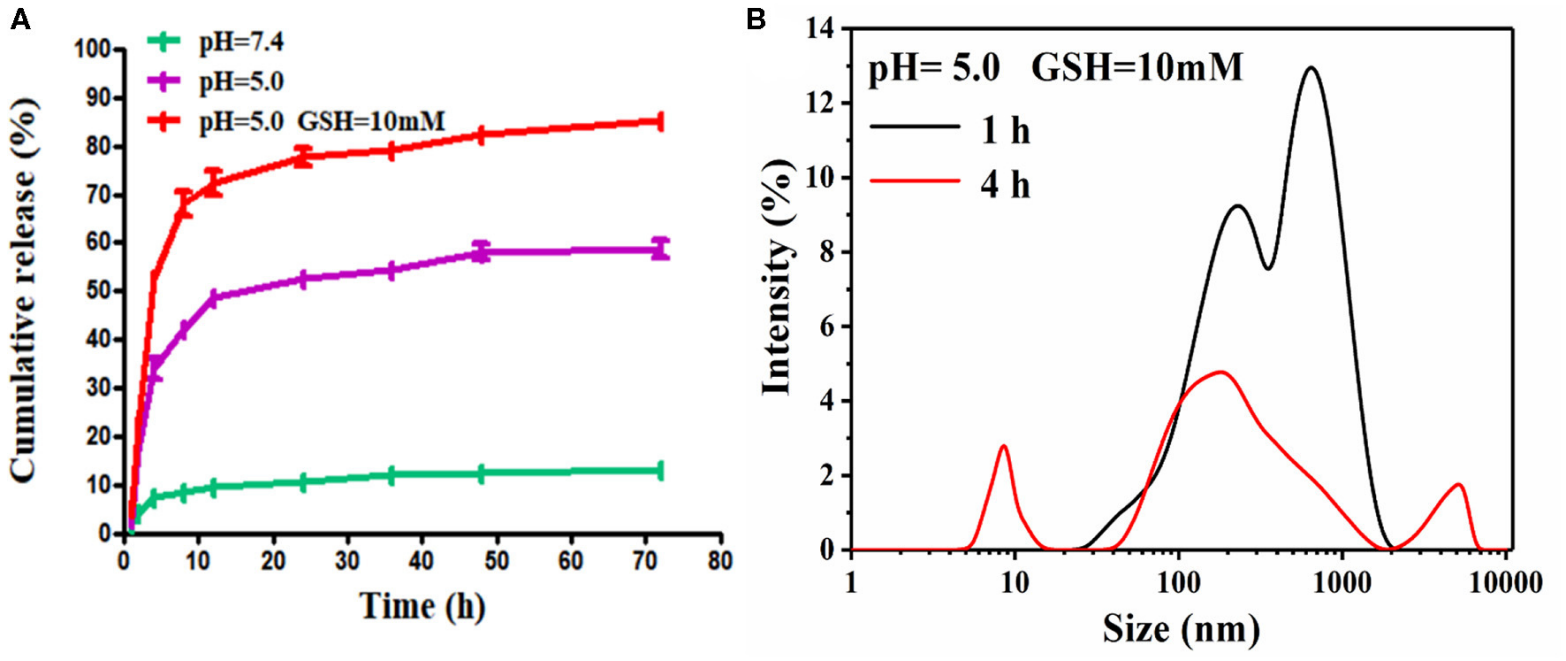
pH and redox dual sensitiveness of HY/SS-CONs. **(A)** Drug release profiles of DOX-loaded HY/SS-CONs under PBS 7.4, PBS pH 5.0, and PBS pH 5.0 with 10 mM GSH; **(B)** Size changes of HY/SS-CONs incubated at pH 5.0 with 10 mM GSH for different time.

The hydrophobic DOX can be efficiently encapsulated into HY/SS-CONs. The drug loading content of HY/SS-CONs was found to be about 18%. The high loading capacity of DOX was due to the fact that HY/SS-CONs had a large pore surface area. Moreover, the other factor can be ascribed to the strong hydrophobic interactions and the π-π stacking interactions between aromatic rings of CONs and DOX. Apparently, this kind of high drug loading content of HY/SS-CONs should be desirable for effective chemotherapy. The drug release from the DOX-loaded HY/SS-CONs were comparatively investigated under PBS 7.4, PBS pH 5.0, and PBS pH 5.0 with 10 mM GSH. The presence of PBS pH 5.0 and 10 mM GSH was used to mimic the tumor intracellular microenvironment. As shown in Figure 4A, the DOX release from the HY/SS-CONs in PBS pH 7.4 was very low. Only about 10% of the total encapsulated DOX was released at 72 h. This may be due to the strong interactions between CONs and DOX as well as the extremely low solubility of DOX at pH 7.4. However, the DOX-loaded HY/SS-CONs exhibited an evidently acid- and GSH-dependent release profile. The DOX release rate in PBS pH 5.0 was much higher than that in PBS pH 7.4, indicating the acid-induced disintegration of HY/SS-CONs, increasing the release rate of DOX. Moreover, the DOX release from HY/SS-CONs in the PBS pH 5.0 with 10 mM GSH was significantly higher than that without GSH. In the presence of PBS pH 5.0 and 10 mM GSH, the cumulative release of DOX can fast achieve about 50% in 4 h and about 90% in 72 h, indicating the acid and GSH dual-triggered disintegration of HY/SS-CONs. To confirm the acid and GSH triggered disintegration, DLS was used to measure the size change of HY/SS-CONs after incubation in PBS pH 5.0 with 10 mm GSH for different time. Compared with the size change of HY/SS-CONs in Figure 3C, it can be found in Figure 4B that a significantly different size distribution of HY/SS-CONs after incubation in PBS pH 5.0 with 10 mm GSH for 1 h was observed. Moreover, after incubation for 4 h, HY/SS-CONs presented a more dramatic fluctuation of size, which can be ascribed to the acid and GSH dual-triggered disassembly and thus the aggregation of HY/SS-CONs fragments due to hydrophobic and π-π stacking interactions. Collectively, the HY/SS-CONs had a very high loading capacity of DOX, but meanwhile possessed a controlled drug release behavior under tumor intracellular microenvironment, which should be very beneficial for cancer treatment.

### *In vitro* Cytotoxicity and Cell Uptake

**Figure 5:**
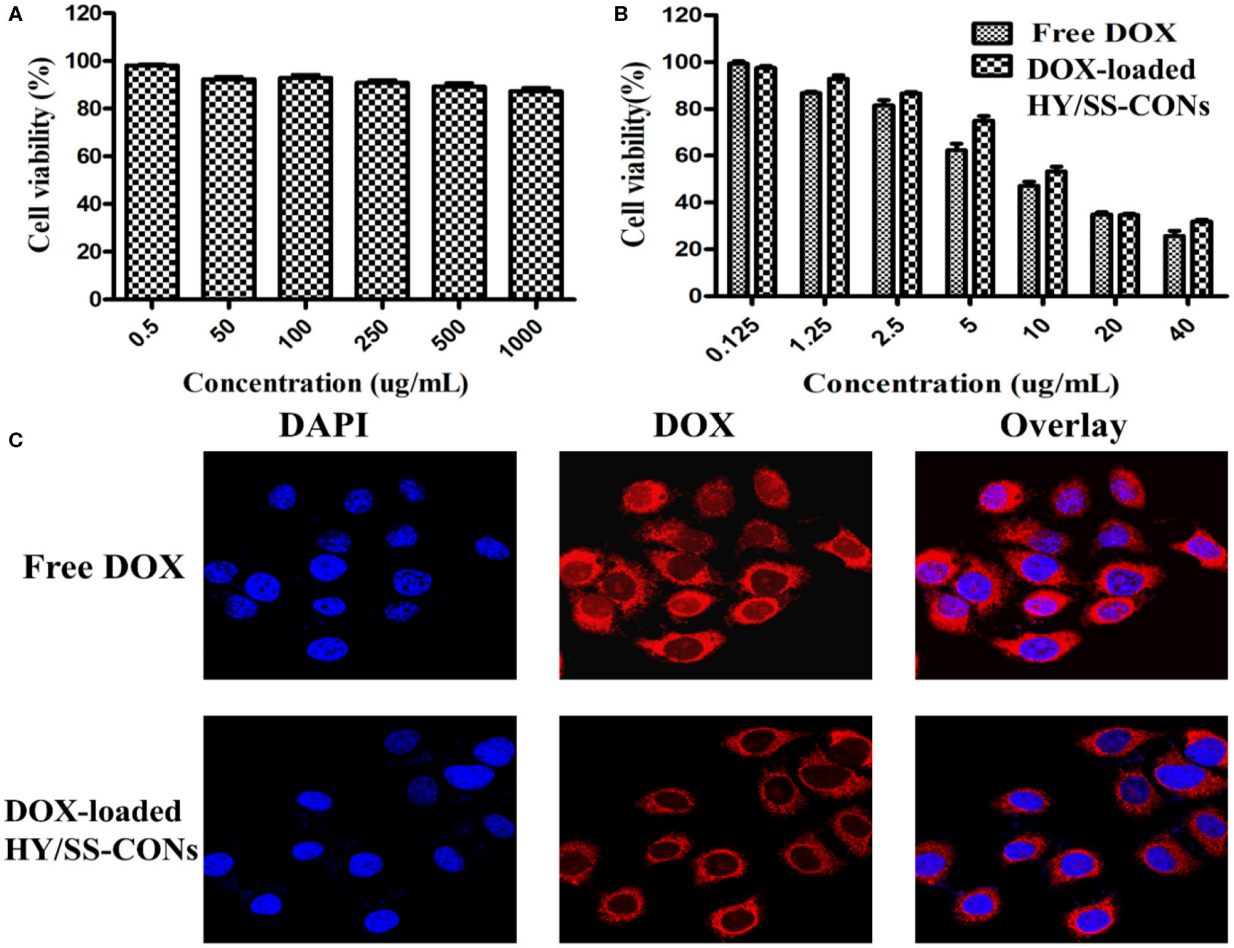
*In vitro* cytotoxicity and cell uptake. **(A)** Viability of HepG2 cells treated with drug-free HY/SS-CONs; **(B)** Cytotoxicity of free DOX and DOX-loaded HY/SS-CONs against HepG2 cells; **(C)** Representative fluorescence microscopy images of HepG2 cells treated with free DOX and DOX-loaded HY/SS-CONs for 4 h.

The viability of HepG2 cells treated with HY/SS-CONs for 24 h was investigated and shown in Figure 5A. HY/SS-CONs had no significant cytotoxicity to the cells. The PEGylated shell coated on the surface of CONs core should contribute the improved biocompatibility. The antitumor activity of free DOX and DOX-loaded HY/SS-CONs was investigated and presented in Figure 5B. The antitumor activity of DOX-loaded HY/SS-CONs exhibited a similar activity with the free DOX. The half inhibitory concentration (IC_50_) values of the DOX-loaded HY/SS-CONs was slightly higher than that of free DOX. Their cell killing activity was in accordance with the cell uptake as demonstrated in Figure 5C. Images of the HepG2 cells incubated with free DOX and DOX-loaded HY/SS-CONs at equivalent DOX concentrations for 4 h. After incubation with free DOX, the strong fluorescence signal in the cell cytoplasm can be observed. The free DOX molecules can rapidly enter cells by a passive diffusion mechanism. The DOX fluorescence intensity for the tumor cells treated with HY/SS-CONs was slightly lower than those treated with free DOX. The nanoscale HY/SS-CONs were internalized through an endocytosis mechanism following an acid- and GSH-triggered fast drug release, thus leading to a similar DOX fluorescence compared with free DOX. Compared with free DOX, the acid and redox dual-sensitive HY/SS-CONs can achieve a very high DOX distribution within tumor cells, which are expected to realize an improved anticancer activity.

## Conclusions

In this work, we designed and prepared a kind of novel PEGylated pH and redox dual-sensitive HY/SS-CONs for effective loading and delivery of DOX. HY/SS-CONs with a PEG shell and a multifunctional core derived from the acid-cleavable hydrazone and redox-responsive disulfide bonds linked COFs. The results indicated that the HY/SS-CONs with well-controlled morphology and good biocompatibility were obtained, which can achieve a high DOX loading content and maintain stability under extracellular condition, but realize intracellular fast drug delivery in response to the low pH and high GSH concentration within tumor cells, leading to an effective antitumor activity to tumor cells. Considering the simple preparation process, high drug loading capacity, and desirable pH and redox dual-responsiveness, the HY/SS-CONs designed herein are expected to serve as a promising nanoplatform for anticancer drug delivery.

## Data Availability Statement

The raw data supporting the conclusions of this article will be made available by the authors, without undue reservation.

## Author Contributions

HL and SL carried out the experiments and drafted the manuscript. ZW participated in the design of the study. CW wrote and revised this paper. JZ supervised the whole study. All authors reviewed the final version of the manuscript and approved it for publication.

## Conflict of Interest

The authors declare that the research was conducted in the absence of any commercial or financial relationships that could be construed as a potential conflict of interest.

## References

[B1] Bai L. S. Z. Phua W. Q. Lim A. Jana Z. Luo H. P. Tham L.. (2016). Nanoscale covalent organic frameworks as smart carriers for drug delivery. Chem. Commun. 52, 4128–4131. 10.1039/C6CC00853D 26877025

[B2] Blanco E. Shen H Ferrari M. (2015). Principles of nanoparticle design for overcoming biological barriers to drug delivery. Nat. Biotechnol. 33, 941–951. 10.1038/nbt.3330 26348965 PMC4978509

[B3] Bozzuto G. Molinari A. (2015). Liposomes as nanomedical devices. Int. J. Nanomed. 10, 975–999. 10.2147/IJN.S68861 25678787 PMC4324542

[B4] Chabner B. A. Roberts T. G. Jr. (2005). Chemotherapy and the war on cancer. Nat. Rev. Cancer 5, 85–98. 10.1038/nrc1529 15630416

[B5] Cheng R. Feng F. Meng F. Deng C. Feijen J. Zhong Z. (2011). Glutathione-responsive nano-vehicles as a promising platform for targeted intracellular drug and gene delivery. J. Control Release 152, 2–12. 10.1016/j.jconrel.2011.01.030 21295087

[B6] Dawidczyk C. M. Russell L. M. Searson P. C. (2014). Nanomedicines for cancer therapy: state-of-the-art and limitations to pre-clinical studies that hinder future developments. Front. Chem. 2:69. 10.3389/fchem.2014.00069 25202689 PMC4142601

[B7] Deng L. Dong H. Dong A. Zhang J. (2015). A strategy for oral chemotherapy via dual pH-sensitive polyelectrolyte complex nanoparticles to achieve gastric survivability, intestinal permeability, hemodynamic stability and intracellular activity. Eur. J. Pharm. Biopharm. 97, 107–117. 10.1016/j.ejpb.2015.10.010 26515259

[B8] Diercks C. S. Yaghi O. M. (2017). The atom, the molecule, and the covalent organic framework. Science 355:eaal1585. 10.1126/science.aal1585 28254887

[B9] Du X. Kleitz F. Li X. Huang H. Zhang X. Qiao S. Z. (2018). Disulfide-bridged organosilica frameworks: designed, synthesis, redox-triggered biodegradation, and nanobiomedical applications. Adv. Func. Mater. 28:1707325 10.1002/adfm.201707325

[B10] Fang Q. Wang J. Gu S. Kaspar R. B. Zhuang Z. Zheng J.. (2015). 3D porous crystalline polyimide covalent organic frameworks for drug delivery. J. Am. Chem. Soc. 137, 8352–8355. 10.1021/jacs.5b04147 26099722

[B11] Hashemzadeh H. Raissi H. (2018). Covalent organic framework as smart and high efficient carrier for anticancer drug delivery: a DFT calculations and molecular dynamics simulation study. J. Phys. D Appl. Phys. 51:345401 10.1088/1361-6463/aad3e8

[B12] Huang H. C. Barua S. Sharma G. Dey S. K. Rege K. (2011). Inorganic nanoparticles for cancer imaging and therapy. J. Control. Release 155, 344–357. 10.1016/j.jconrel.2011.06.004 21723891

[B13] Kamaly N. Yameen B. Wu J. Farokhzad O. C. (2016). Degradable controlled-release polymers and polymeric nanoparticles: mechanisms of controlling drug release. Chem. Rev. 116, 2602–2663. 10.1021/acs.chemrev.5b00346 26854975 PMC5509216

[B14] Kandambeth S. Dey K. Banerjee R. (2018). Covalent organic frameworks: chemistry beyond the structure. J. Am. Chem. Soc. 141, 1807–1822. 10.1021/jacs.8b10334 30485740

[B15] Lee M. H. Yang Z. Lim C. W. Lee Y. H. Dongbang S. Kang C.. (2013). Disulfide-cleavage-triggered chemosensors and their biological applications. Chem. Rev. 113, 5071–5109. 10.1021/cr300358b 23577659

[B16] Liu F. Lin L. Zhang Y. Sheng S. Wang Y. Xu C.. (2019). Two-dimensional nanosheets with high curcumin loading content for multimodal imaging-guided combined chemo-photothermal therapy. Biomaterials 223:119470. 10.1016/j.biomaterials.2019.119470 31526950

[B17] Liu H. Shi X. Wu D. Khshen F. K. Deng L. Dong A.. (2019). Injectable, biodegradable, thermosensitive nanoparticles-aggregated hydrogel with tumor-specific targeting, penetration, and release for efficient postsurgical prevention of tumor recurrence. ACS Appl. Mater. Interfaces 11, 19700–19711. 10.1021/acsami.9b01987 31070356

[B18] Liu S. Yang J. Guo R. Deng L. Dong A. Zhang J. (2020). Facile fabrication of redox-responsive covalent organic framework nanocarriers for efficiently loading and delivering doxorubicin. Macromol. Rapid Commun. 41:1900570. 10.1002/marc.201900570 31894599

[B19] Ma H. Liu B. Li B. Zhang L. Li Y. G. Tan H. Q.. (2016). Cationic covalent organic frameworks: a simple platform of anionic exchange for porosity tuning and proton conduction. J. Am. Chem. Soc. 138, 5897–5903. 10.1021/jacs.5b13490 27094048

[B20] Ma J. Deng H. Zhao F. Deng L. Wang W. Dong A.. (2018). Liposomes-camouflaged redox-responsive nanogels to resolve the dilemma between extracellular stability and intracellular drug release. Macromol. Biosci. 18:201800049. 10.1002/mabi.201800049 29732706

[B21] Mitra S. Sasmal H. S. Kundu T. Kandambeth S. Illath K. Diaz Diaz D.. (2017). Targeted drug delivery in covalent organic nanosheets (CONs) via sequential postsynthetic modification. J. Am. Chem. Soc. 139, 4513–4520. 10.1021/jacs.7b00925 28256830

[B22] Mura S. Nicolas J. Couvreur P. (2013). Stimuli-responsive nanocarriers for drug delivery. Nat. Mater. 12, 991–1003. 10.1038/nmat3776 24150417

[B23] Raemdonck K. Braeckmans K. Demeester J. De Smedt S. C. (2014). Merging the best of both worlds: hybrid lipid-enveloped matrix nanocomposites in drug delivery. Chem. Soc. Rev. 43, 444–472. 10.1039/C3CS60299K 24100581

[B24] Sakaushi K. Antonietti M. (2015). Carbon- and nitrogen-based organic frameworks. Acc. Chem. Res. 48, 1591–1600. 10.1021/acs.accounts.5b00010 26000989

[B25] Segura J. L. Mancheno M. J. Zamora F. (2016). Covalent organic frameworks based on schiff-base chemistry: synthesis, properties and potential applications. Chem. Soc. Rev. 45, 5635–5671. 10.1039/C5CS00878F 27341661

[B26] Shi J. Kantoff P. W. Wooster R. Farokhzad O. C. (2017). Cancer nanomedicine: progress, challenges and opportunities. Nat. Rev. Cancer 17, 20–37. 10.1038/nrc.2016.108 27834398 PMC5575742

[B27] Song Y. Sun Q. Aguila B. Ma S. (2019). Opportunities of covalent organic frameworks for advanced applications. Adv. Sci. 6:1801410. 10.1002/advs.201801410 30693185 PMC6343072

[B28] Vines J. B. Yvon J. H. Ryu N. E. Lim D. J. Park H. (2019). Gold nanoparticles for photothermal cancer therapy. Front. Chem. 7:167. 10.3389/fchem.2019.00167 31024882 PMC6460051

[B29] Vyas V. S. Vishwakarma M. Moudrakovski I. Haase F. Savasci G. Ochsenfeld C.. (2016). Exploiting noncovalent interactions in an imine-based covalent organic framework for quercetin delivery. Adv. Mater. 28, 8749–8754. 10.1002/adma.201603006 27545588

[B30] Waller P. J. Gandara F. Yaghi O. M. (2015). Chemistry of covalent organic frameworks. Acc. Chem. Res. 48, 3053–3063. 10.1021/acs.accounts.5b00369 26580002

[B31] Wang K. Zhang Z. Lin L. Chen J. Hao K. Tian H. (2019). Covalent organic nanosheets integrated heterojunction with two strategies to overcome hypoxic-tumor photodynamic therapy. Chem. Mater. 31, 3313–3323. 10.1021/acs.chemmater.9b00265

[B32] Wang S. B. Chen Z. X. Gao F. Zhang C. Zou M. Z. Ye J. J.. (2020). Remodeling extracellular matrix based on functional covalent organic framework to enhance tumor photodynamic therapy. Biomaterials 234:119772. 10.1016/j.biomaterials.2020.119772 31945618

[B33] Wu M. X. Yang Y. W. (2017). Applications of covalent organic frameworks (COFs): from gas storage and separation to drug delivery. Chinese Chem. Lett. 28, 1135–1143. 10.1016/j.cclet.2017.03.026

[B34] Xiong S. Wang Y. Wang X. Chu J. Zhang R. Gong M. (2020). Schiff base type conjugated organic framework nanofibers: solvothermal synthesis and electrochromic properties. Sol. Energ. Mat. Sol. C 209:110438 10.1016/j.solmat.2020.110438

[B35] Xu C. Lei C. Yu C. Z. (2019). Mesoporous silica nanoparticles for protein protection and delivery. Front. Chem. 7:290. 10.3389/fchem.2019.00290 31119124 PMC6504683

[B36] Yu L. Dong A. Guo R. Yang M. Deng L. Zhang J. (2018). DOX/ICG coencapsulated liposome-coated thermosensitive nanogels for NIR-triggered simultaneous drug release and photothermal effect. ACS Biomater. Sci. Eng. 4, 2424–2434. 10.1021/acsbiomaterials.8b00379 33435106

[B37] Zhang G. Li X. Liao Q. Liu Y. Xi K. Huang W.. (2018). Water-dispersible PEG-curcumin/amine-functionalized covalent organic framework nanocomposites as smart carriers for *in vivo* drug delivery. Nat. Commun. 9:2785. 10.1038/s41467-018-04910-5 30018290 PMC6050241

[B38] Zhang H. Li Q. Liu R. Zhang X. Li Z. Luan Y. (2018). A versatile prodrug strategy to *in situ* encapsulate drugs in MOF nanocarriers: a case of cytarabine-IR820 prodrug encapsulated ZIF-8 toward chemo-photothermal therapy. Adv. Funct. Mater. 28:1802830 10.1002/adfm.201802830

[B39] Zhang J. Gao W. Fang R. H. Dong A. Zhang L. (2015). Synthesis of nanogels via cell membrane-templated polymerization. Small 11, 4309–4313. 10.1002/smll.201500987 26044721 PMC4562875

[B40] Zhang J. Lin X. Liu J. Zhao J. Dong H. Deng L.. (2013). Sequential thermo-induced self-gelation and acid-triggered self-release process of drug-conjugated nanoparticles: a strategy for the sustained and controlled drug delivery to tumors. J. Mater. Chem. B 1, 4667–4677. 10.1039/c3tb20597e 32261210

[B41] Zhao F. Dong A. Deng L. Guo R. Zhang J. (2019). Morphology control and property design of boronate dynamic nanostructures. Polym. Chem. 10, 2436–2446. 10.1039/C9PY00217K

[B42] Zhao F. Liu H. Mathe S. D. R. Dong A. Zhang J. (2018). Covalent organic frameworks: from materials design to biomedical application. Nanomaterials 8:15. 10.3390/nano8010015 29283423 PMC5791102

[B43] Zhao X. Deng L. Deng H. Dong A. Wang W. Zhang J. (2018). *In situ* template polymerization to prepare liposome-coated PDMAEMA nanogels with controlled size, high stability, low cytotoxicity, and responsive drug release for intracellular dox release. Macromol. Chem. Physi. 219:201800071 10.1002/macp.201800071

